# Didemnaketals F and G, New Bioactive Spiroketals from a Red Sea Ascidian *Didemnum* Species

**DOI:** 10.3390/md12095021

**Published:** 2014-09-25

**Authors:** Lamiaa A. Shaala, Diaa T.A. Youssef, Sabrin R.M. Ibrahim, Gamal A. Mohamed, Jihan M. Badr, April L. Risinger, Susan L. Mooberry

**Affiliations:** 1 Natural Products Unit, King Fahd Medical Research Center, King Abdulaziz University, Jeddah 21589, Kingdom of Saudi Arabia; E-Mail: lshalla@kau.edu.sa; 2 Suez Canal University Hospital, Suez Canal University, Ismailia 41522, Egypt; 3 Department of Natural Products, Faculty of Pharmacy, King Abdulaziz University, Jeddah 21589, Kingdom of Saudi Arabia; E-Mail: dyoussef@kau.edu.sa; 4 Department of Pharmacognosy, Faculty of Pharmacy, Suez Canal University, Ismailia 41522, Egypt; E-Mail: jihanbadr2010@hotmail.com; 5 Department of Pharmacognosy, Faculty of Pharmacy, Assiut University, Assiut 71526, Egypt; E-Mail: sabrinshaur@gmail.com; 6 Department of Pharmacognosy and Medicinal Chemistry, Faculty of Pharmacy, Taibah University, Al Madinah Al Munawwarah 3001, Kingdom of Saudi Arabia; 7 Department of Pharmacognosy, Faculty of Pharmacy, Al-Azhar University, Assiut Branch, Assiut 71524, Egypt; E-Mail: gamals2001@yahoo.com; 8 Department of Pharmacology and Cancer Therapy & Research Center, University of Texas Health Science Center at San Antonio, San Antonio, TX 78229, USA; E-Mails: risingera@uthscsa.edu (A.L.R.); mooberry@uthscsa.edu (S.L.M.)

**Keywords:** Red Sea ascidian, *Didemnum* species, spiroketals, didemnaketals F and G, HeLa cells, antiproliferative and cytotoxic activity, antimicrobial activity

## Abstract

In continuation of our ongoing efforts to identify bioactive compounds from Red Sea marine organisms, a new collection of the ascidian *Didemnum* species was investigated. Chromatographic fractionation and HPLC purification of the CH_2_Cl_2_ fraction of an organic extract of the ascidian resulted in the identification of two new spiroketals, didemnaketals F (**1**) and G (**2**). The structure determination of the compounds was completed by extensive study of 1D (^1^H, ^13^C, and DEPT) and 2D (COSY, HSQC, and HMBC) NMR experiments in addition to high-resolution mass spectral data. Didemnaketal F (**1**) and G (**2**) differ from the previously reported compounds of this class by the lack the terminal methyl ester at C-1 and the methyl functionality at C-2. Instead, **1** and **2** possess a methyl ketone moiety instead of the terminal ester. Furthermore, didemnaketal F possesses a disubstituted double bond between C-2 and C-3, while the double bond was replaced by a secondary alcohol at C-3 in didemnaketal G. In addition, they possess the unique spiroketal/hemiketal functionality which was previously reported in didemnaketal E. Didemnaketals F (**1**) and G (**2**) displayed moderate activity against HeLa cells with of IC_50s_ of 49.9 and 14.0 µM, respectively. In addition, didemnaketal F (**1**) displayed potent antimicrobial activity against *E. coli* and *C. albicans.* These findings provide further insight into the biosynthetic capabilities of this ascidian and the chemical diversity as well as the biological activity of this class of compounds.

## 1. Introduction

Natural products and their derivatives contribute more than half of all clinically administered drugs [1]. Less than 1% of the isolated marine-derived natural products have been screened for their bioactivities [2]. Many marine invertebrates such as sponges and ascidians (tunicates) are sessile (attached to the ocean floor) and use highly evolved chemical compounds to attract food, block the growth of intruding neighbors or repel predators. Biologists believe that these survival demands triggered the evolution of a particularly abundant mixture of bioactive compounds. Marine invertebrates, including sponges and ascidians, contribute a major proportion of novel marine-derived bioactive compounds [3]. The search for the bioactive compounds from marine ascidians is relatively young, but it is worthwhile mentioning that didemnin B, an ascidian metabolite, was the first marine natural product to be evaluated in a human clinical trial [4]. Most ascidians are consumed as food in some countries. They produce antifouling natural products and this can be considered as a kind of autogenic protection [5]. The genus *Didemnum* is well-known for its diverse and bioactive secondary metabolites including cyclic peptides [6,7], alkaloids [8,9,10,11,12], macrolides [13,14,15], and *N*,*N′*-diphenethylurea [16]. These metabolites were found to possess different biological activities such as antiplasmodial [17], antiviral [18,19], cytotoxic [20], and protein kinase inhibitors [13,14,15]. It is well-known that the first marketed marine-derived anticancer agent, trabectedin (ET-743, Yondelis), is a product of the tunicate *Ecteinascidia turbinata* [21]. It is commercialized by PharmaMar and is approved for the treatment of soft tissue sarcomas in Europe and other countries. It is also undergoing clinical trials for the treatment of locally advanced or metastatic ovarian cancer, liposarcoma, leiomyosarcoma or pleural mesothelioma [22].

**Figure 1:**
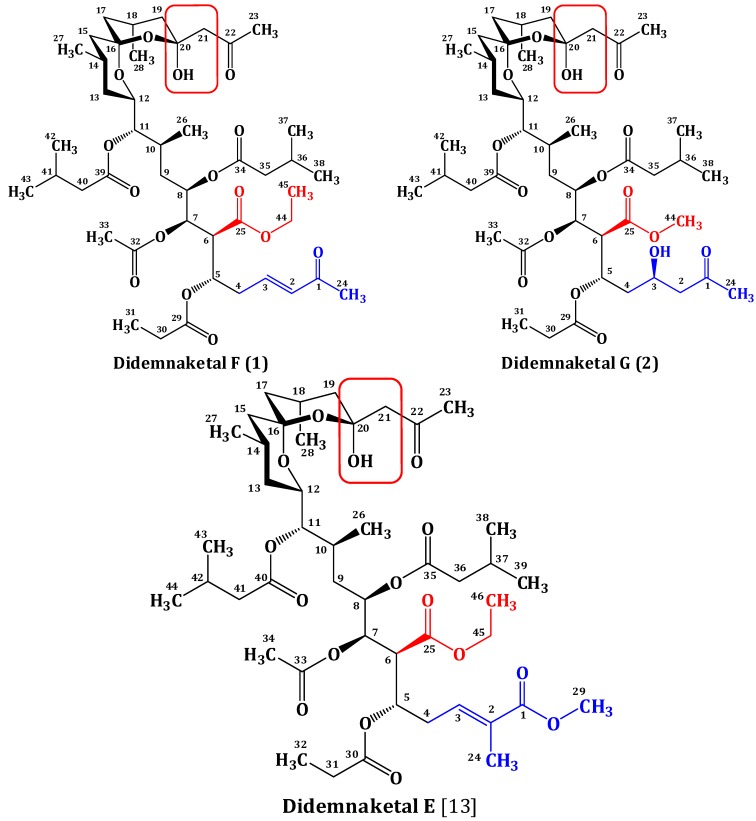
Structures of didemnaketals F (**1**), G (**2**), and E with key moieties and differences.

To date, five spiroketals, including didemnaketals A–E, were isolated from the marine ascidian *Didemnum* species collected from different geographical locations including the Red Sea [13] and Palau [14,15]. Two of these, didemnaketals A and B, were reported as a result of oxidation and methanolysis of didemnaketal C during long storage of the ascidian sample in MeOH [14]. In this work, we describe the isolation, structure determination, and biological activities of two didemnaketal congeners, didemnaketal F (**1**) and G (**2**) (Figure 1) from a new collection of the Red Sea *Didemnum* species. The new compounds differ from the previously reported ones in which they lack the methyl functionality at C-2 and the terminal methyl ester moiety of the spiroketal skeleton. Instead of the terminal methyl ester, they possess a terminal methyl ketone moiety at the left part of the molecule (as in **1** and **2**). Additionally, compound **1** possesses a disubstituted double bond at C-2/C-3, while **2** possesses a secondary alcohol at C-3. As reported before in didemnaketal E (Figure 1) [13], both **1** and **2** possess a spiroketal/hemiketal functionality at the right part of the molecule.

## 2. Results and Discussion

### 2.1. Purification of Compounds **1** and **2**

Investigation of the CH_2_Cl_2_ soluble fraction of an organic extract of a new collection of the Red Sea ascidian *Didemnum* species using a combination of chromatographic techniques, including normal SiO_2_, Sephadex LH-20, and C18 HPLC, afforded two new spiroketals; didemnaketals F (**1**) and G (**2**) (Figure 1). The isolated compounds were evaluated for their antiproliferative, cytotoxic, and antimicrobial activities.

### 2.2. Structure Elucidation of Compound **1**

**Figure 2:**
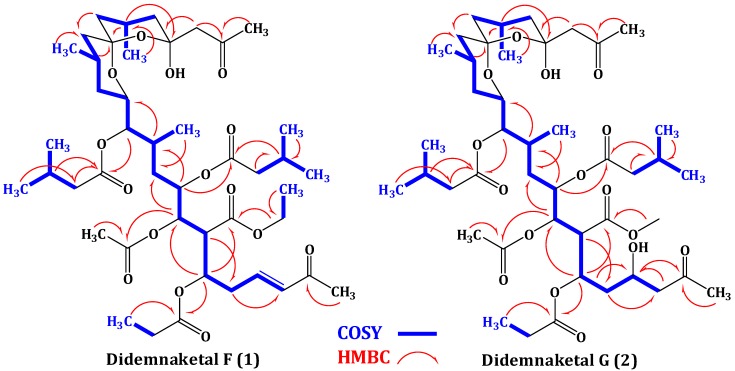
Key COSY and HMBC correlations didemnaketals F (**1**) and G (**2**).

Compound **1** possesses a molecular formula C_45_H_72_O_15_ as deduced from the HRESIMS molecular ion peak at *m*/*z* 853.4950 [M + H]^+^, suggesting 10 degrees of unsaturation. Compound **1** is 30 mass units (CH_2_O) less than didemnaketal E (Figure 1) [13]. The IR spectrum of **1** displayed characteristic absorption bands for the functional groups in the molecule at 3460 (OH), 1735 (ester), 1720 (ketone), 1675 (α,β-unsaturated ketone) and 1650 (C=C), 1070 (C-O) cm^−1^. The ^13^C NMR, DEPT, and HSQC spectra revealed the presence of 45 carbon resonances: 12 methyls, 11 methylenes, 13 methines, five of them were assigned to oxymethines and two for disubstituted olefinic double bond, and 9 quaternary carbons including five ester and two ketone carbonyls. The presence of the unique spiroketal/hemiketal moiety was evident from carbon signals at δ_C_ 98.7 (C-16) and 99.8 (C-20) [13]. The ^1^H-^1^H COSY spectrum revealed the presence of several spin systems (Table 1, Figure 2). These spin systems were confirmed by the HSQC and HMBC cross peaks. The ^1^H and ^13^C NMR data of **1** were comparable to those of didemnaketal E in the C4C23 region [13]. The differences in the NMR spectra of **1** were the absence of the signals associated with the methyl group at C-2 and the terminal methyl ester moiety in didemnaketal E (Figure 1) [13]. Instead, the ^1^H and ^13^C NMR spectra of **1** showed signals at δ_H_ 6.11 (d, *J* = 16.5 Hz, H-2)/δ_C_ 133.0 (C-2) and 6.75 (dt, *J* = 16.5, 6.8 Hz, H-3)/δ_C_ 145.8 (C-3). These signals were assigned for a disubstituted double bond and confirmed by the observed COSY correlations between H-2 and H-3 and between H-3 and H_2_-4 (δ_H_ 1.97, m). The *E*-configured double bond was confirmed by a large ^3^*J*_H_,_H_ value (^3^*J*_H-2,H-3_ = 16.5 Hz) [23]. The position of the double bond at C-2 and C-3 was established by the COSY correlation between H-3 and H_2_-4 (δ_H_ 1.97, m) and further secured by the ^3^*J* HMBC correlation between H_2_-4 and C-2. Moreover, signals for a terminal methyl ketone at δ_H_ 2.24 (s, H_3_-24)/δ_C_ 27.1 (C-24) and δ_C_ 198.5 (C-1) were observed. This was confirmed by the ^2^*J* HMBC cross peak between H_3_-24 and C-1 (δ_C_ 198.5). The upfield shift of the ketone moiety at C-1 (δ_C_ 198.5) supported the α,β-unsaturation at C-2/C-3. The ^13^C chemical shift values of C-2 and C-3 and the HMBC cross peak of H-3 with C-1 confirmed the connectivity of methyl ketone moiety at C-2. Furthermore, the ^1^H and ^13^C NMR spectra showed the presence of one acetate at δ_H_ 2.05 (H_3_-33)/δ_C_ 21.0 (C-33) and 170.2 (C-32), one ethoxy at δ_H_ 4.11 (H_2_-44)/δ_C_ 60.4 (C-44) and δ_H_ 1.27 (H_3_-45)/δ_C_ 14.3 (C-45), one propionate at δ_H_ 2.16 (H_2_-30)/δ_C_ 26.8 (C-30), δ_H_ 1.03 (H_3_-31)/δ_C_ 7.5 (C-31), and δ_C_ 170.5 (C-29), two isovalerate moieties at δ_H_ 2.13 (H_2_-35)/δ_C_ 41.8 (C-35), δ_H_ 2.87 (H-36)/δ_C_ 33.5 (C-36), δ_H_ 1.20 (H_3_-37, H_3_-38)/δ_C_ 17.2 (C-37, C-38), and δ_C_ 176.6 (C-34) and δ_H_ 2.03 (H_2_-40)/δ_C_ 32.7 (C-40), δ_H_ 1.97 (H-41)/δ_C_ 30.1 (C-41), δ_H_ 0.95 (H_3_-42, H_3_-43)/δ_C_ 22.0 (C-42, C-43), and δ_C_ 168.6 (C-39), and one methyl ketone at δ_H_ 2.19 (H_3_-23)/δ_C_ 31.1 (C-23) and δ_C_ 202.5 (C-22). These moieties were confirmed by the observed COSY and HMBC correlations (Table 1, Figure 2). Their positions at C-7, C-25, C-5, C-8, C-11, and C-21, respectively were defined by the HMBC correlations: H-7 to C-32, H-44 to C-25, H-5 to C-29, H-8 to C-34, H-11 to C-39, and H-21 to C-22 (Figure 2, Table 1). The absolute configuration of **1** was assigned by comparison of ^1^H and ^13^C NMR data as well as coupling constants with those of previously reported didemnaketals [13,14,15,24,25,26]. From the above discussion and the results of the COSY, HSQC, and HMBC (Table 1, Figure 2) spectra, the structure of **1** was unambiguously assigned as shown and named didemnaketal F.

**Table 1:** NMR data of didemnaketal F (**1**) (CDCl_3_, 600 and 150 MHz).

No.	δ_C_, m ^a^	δ_H_ m (*J* in Hz)	^1^H-^1^H COSY	HMBC
1	198.5, qC	-	-	-
2	133.0, CH	6.11 d (16.5)	3	-
3	145.8, CH	6.75 dt (16.5, 6.8)	2, 4	1
4	29.9, CH_2_	1.97 m	3, 5	2, 6, 25
5	72.3, CH	3.45dt (11.6, 7.3)	4, 6	6, 7, 25, 29
6	41.6, CH	2.30 dt (11.6, 6.9)	5, 7	4, 25, 32
7	67.9, CH	4.83 t (9.6)	6, 8	5, 9, 32
8	67.5, CH	4.30 m	7, 9	6, 7, 10, 34
9	36.8, CH_2_	1.70 m, 1.25 m	8, 10	7, 8, 10
10	34.1, CH	1.92 m	9, 26	8, 12
11	76.5, CH	5.02 dd (6.8, 6.3)	10, 12	9, 12, 39
12	75.3, CH	3.86 m	11, 13	10, 11, 16
13	36.1, CH_2_	1.48 m, 0.88 m	12, 14	11, 14, 15
14	25.5, CH	1.72 m	13, 15	12, 16
15	44.8, CH_2_	1.62 m, 1.08 m	14	16, 17
16	98.7, qC	-	-	-
17	42.0, CH_2_	1.72 m, 1.56 m	18	15, 16
18	24.6, CH	1.75 m	17, 19, 28	16, 20
19	33.1, CH_2_	2.15 m, 1.74 m	18	17, 20
20	99.8, qC	-	-	-
21	41.2, CH_2_	2.95 m, 2.86 m	-	20, 22
22	202.5, qC	-	-	-
23	21.3, CH_3_	2.19 s	-	22
24	29.7, CH_3_	2.24 s	-	1
25	172.6, qC	-	-	-
26	14.8, CH_3_	0.90 d (6.5)	10	9, 10
27	22.2, CH_3_	0.91 d (6.8)	14	13, 14, 15, 16
28	19.8, CH_3_	0.97 d (6.5)	18	18, 19, 20
29	170.5, qC	-	-	-
30	26.6, CH_2_	2.16 q (7.3)	31	29, 31
31	7.5, CH_3_	1.03 t (7.3)	30	29, 30
32	170.2, qC	-	-	-
33	21.0, CH_3_	2.05 s	-	32
34	176.6, qC	-	-	-
35	41.8, CH_2_	2.13 m	36	37, 38
36	33.9, CH	2.87 m	35, 37, 38	34, 35
37	17.2, CH_3_	1.20 d (6.8)	36	34, 35, 36
38	17.2, CH_3_	1.20 d (6.8)	36	34, 35, 36
39	168.6, qC	-	-	-
40	33.2, CH_2_	2.03 m	41	39, 41
41	31.2, CH	1.97 m	40, 42	39, 42, 43
42	22.0, CH_3_	0.95 d (6.3)	41	40, 41, 43
43	22.0, CH_3_	0.95 d (6.3)	41	40, 41, 42
44	60.4, CH_2_	4.11 q (6.8)	45	25, 45
45	14.3, CH_3_	1.27 t (6.8)	44	44

^a^ Multiplicities were deduced by DEPT and HSQC; CH_3_ = methyl, CH_2_ = methylene, CH = methine, qC = quaternary carbon.

The molecular formula of compound **2** was deduced as C_44_H_72_O_16_ as established from the HRESIMS pseudomolecular ion peak at *m*/*z* 857.4899 [M + H]^+^, suggesting nine degrees of unsaturation. These degrees of unsaturation were attributed to seven carbonyl moieties and two were accounted for the spiroketal/hemiketal moiety. The IR spectrum showed absorption bands at 3465 (OH), 1731 (ester), 1718 (ketone), and 1065 (C-O) cm^−1^. The ^1^H and ^13^C NMR spectral data of **2** were comparable with those of **1** with few exceptions. The signals of H-2/C-2 and H-3/C-3 associated with the olefinic moiety in **1** were absent in **2**. Instead, new signals in **2** for a methylene (H_2_-2) and an oxygenated methine (H-3) were observed at δ_H_ 2.61 (dd, *J* = 14.5, 7.3 Hz, H-2a) and 2.45 (dd, *J* = 14.5, 7.5 Hz, H-2b)/δ_C_ 50.1 (C-2) and δ_H_ 4.28 (quin, *J* = 7.3 Hz, H-3)/δ_C_ 67.5 (C-3). In addition, the downfield shift of the ketone carbonyl at δ_C_ 207.2 (C-1) in **2** compared to δ_C_ 198.5 in **1** suggested the absence of the olefinic moietyin **2**. The ^1^H-^1^H COSY correlations from H_2_-2 to H-3 and from H-3 to H_2_-4, along with HMBC cross-peaks of H_2_-2/C-3 and H_2_-2/C-4, H-3/C-2, H_2_-4/C-2 and H_2_-4/C-3 and H-5/C-3 confirmed the position of the secondary alcohol functionality at C-3. Moreover, the ^1^H and ^13^C NMR spectra of **2** lack the resonating signals associated with the ethoxy moiety at C-25 in **1**. Instead, signals for a methoxy group at δ_H_ 3.64 (H_3_-44)/δ_C_ 51.8 (C-44) were observed in **2**. The placement of methoxy group at C-25 in **2** was confirmed by the HMBC correlation between H_3_-44 and C-25 (Figure 2). The assignment of all quaternary carbons were unambiguously assigned from HMBC correlation (Table 2 and Figure 2) completing the assignment of **2** and was named as didemnaketal G.

The absolute configuration of the previously reported didemnaketals from the genus *Didemnum* was assigned on the basis of degradation/derivatization experiments, chiral shift methods, and chemical synthesis of the C9-C28 subunit [24,25,26]. Recently, the absolute configuration of the C_10_-C_20_ subunit was revised by Fuwa *et al.* [27]. Thus, the absolute configuration of didemnaketals F (**1**) and G (**2**) was assigned as 5*S*, 6*S*, 7*R*, 8*R*, 10*S*, 11*S*, 12*S*, 14*R*, 16*S*, 18*R* based on extensive spectroscopic studies, in addition to comparison with the previously reported didemnaketals.

**Table 2:** NMR data of didemnaketal G (**2**) (CDCl_3_, 600 and 150 MHz).

No.	δ_C_, m ^a^	δ_H_ m (*J* in Hz)	^1^H-^1^H COSY	HMBC
1	207.2, qC	-	-	-
2	50.1, CH_2_	2.61 dd (14.5, 7.3) 2.45 dd (14.5, 7.5)	3	1, 3, 4
3	67.5, CH	4.28 quin (7.3)	2, 4	1, 2
4	36.1, CH_2_	1.65 m, 0.87 m	3, 5	2, 3, 5
5	72.3, CH	3.45dt (11.5, 6.9)	4, 6	3, 6, 7, 25, 29
6	41.7, CH	2.95 dt (11.6, 6.5)	5, 7	4, 8, 25
7	67.9, CH	4.83 t (9.8)	6, 8	5, 9, 32
8	67.5, CH	3.90 m	7, 9	6, 7, 9, 34
9	36.9, CH_2 _	1.75 m, 1.29 m	8, 10	7, 8, 10, 26
10	33.9, CH	1.95 m	9, 11, 26	8, 11, 26, 39
11	76.8, CH	5.01 dd (6.5, 6.3)	10, 12	9, 10, 12, 39
12	75.3, CH	3.86 m	11, 13	10, 14, 16
13	36.1, CH_2_	1.47 m, 0.85 m	12, 14	11, 15
14	26.6, CH	1.70 m	13, 15	12, 16
15	44.7,CH_2_	1.63 m, 1.08 m	14	13, 16
16	98.7, qC	-	-	-
17	41.1, CH_2_	1.74 m, 1.55 m	18	15, 16, 19
18	24.6, CH	1.75 m	17, 19, 28	16, 20, 28
19	32.0, CH_2_	2.15 m	18	20, 21
20	99.8, qC	-	-	-
21	41.1, CH_2_	2.85 m, 2.72 m	-	20, 22, 23
22	202.5, C	-	-	-
23	21.0, CH_3_	2.06 s	-	22
24	31.2, CH_3_	2.16 s	-	1, 2
25	175.8, qC	-	-	-
26	14.8, CH_3_	0.88 d (6.5)	10	9, 10, 11
27	22.0, CH_3_	0.92 d (6.8)	14	12, 13, 14
28	20.5, CH_3_	0.90 d (6.5)	18	17, 18, 19, 20
29	170.2, qC	-	-	-
30	26.8, CH_2_	2.18 q (7.3)	31	29, 31
31	7.5, CH_3_	1.03 t (6.9)	30	29, 30
32	170.5, qC	-	-	-
33	21.3, CH_3_	2.01 s	-	32
34	176.6, qC	-	-	-
35	39.8, CH_2_	2.33 m	36	34, 37, 38
36	33.2, CH	2.85 m	35, 37, 38	34
37	17.2, CH_3_	1.22 d (6.8)	36	34, 35, 36
38	17.2, CH_3_	1.22 d (6.8)	36	34, 35, 36
39	168.6, qC	-	-	-
40	33.1, CH_2_	2.05 m	41	39, 41
41	29.7, CH	1.97 m	40, 42	39, 42, 43
42	22.2, CH_3_	0.93 d (6.3)	41	40, 41, 43
43	22.2, CH_3_	0.93 d (6.3)	41	40, 41, 42
44	51.8, CH_3_	3.64 s	-	25

^a^ Multiplicities were deduced by DEPT and HSQC; CH_3_ = methyl, CH_2_ = methylene, CH = methine, qC = quaternary carbon.

**Figure 3:**
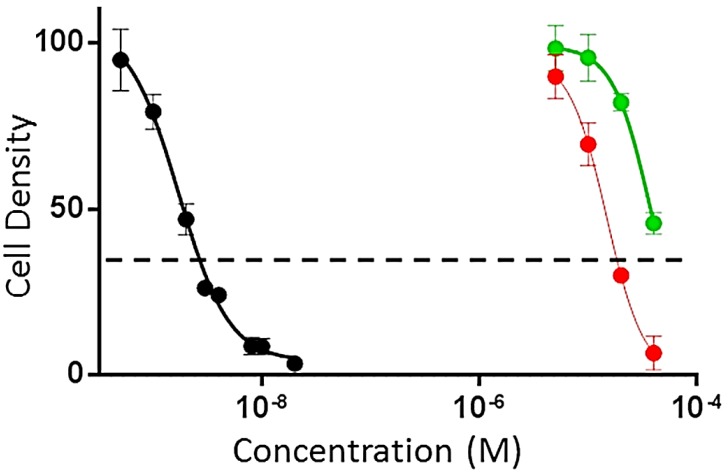
Antiproliferative and cytotoxic activities of didemnaketals F (**1**) (Green), and G (**2**) (Red), and the positive control paclitaxel (Black) in HeLa cells. Dashed line indicates the density of cells at the time of drug addition.

Didemnaketals F (**1**) and G (**2**) were evaluated for their antiproliferative and cytotoxic activities against HeLa cells [28,29,30] as well as antimicrobial activity against three pathogens including a Gram-positive bacterium (*Staphylococcus aureus* ATCC 25923), a Gram-negative bacterium (*Escherichia coli* ATCC 25922) and yeast (*Candida albicans* ATCC 14053). Didemnaketals F (**1**) and G (**2**) displayed moderate antiproliferative activity against HeLa cells with IC_50s_ of 49.9 and 14.0 µM, respectively (Figure 3). Didemnaketal G (**2**) also caused cytotoxicity as indicated by the dose response curve dropping below the dashed line (Figure 3). In contrast, cytotoxic effects were not observed with didemnaketal F (**1**) at concentrations up to 50 μM (Figure 3). Paclitaxel was used as a positive control with an IC_50_ of 0.0017 µM in this assay.

**Figure 4:**
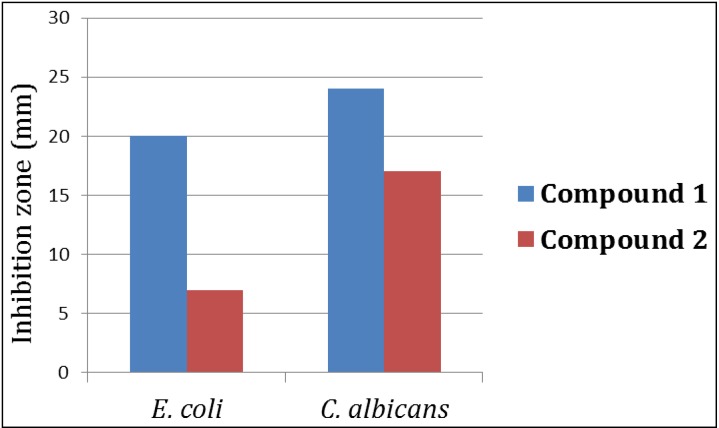
Antimicrobial activities of Didemnaketals F (**1**) and G (**2**) against *E. coli* and *C. albicans*.

In the antimicrobial screening, didemnaketal F (**1**) showed strong antimicrobial activity against *E. coli* and *C. albicans* with inhibition zones of 20 and 24 mm at a concentration of 100 µg/disc, while didemnaketal G (**2**) showed moderate activity against *E. coli* and *C. albicans* with inhibition zones of 7 and 17 mm at the same concentration (Figure 4).

## 3. Experimental Section

### 3.1. General Experimental Procedures

Optical rotations were measured on a JASCO DIP-370 digital polarimeter at 25 °C at the sodium D line (589 nm). UV spectrum was recorded on a Hitachi 300 spectrometer. The IR spectra were measured on a Shimadzu Infrared-400 spectrophotometer (Kyoto, Japan). NMR spectra were determined on BRUKER AVANCE III 600 instruments (600 MHz for ^1^H and 150 MHz for ^13^C NMR). NMR chemical shifts are expressed in parts per million (ppm) referenced to residual CHCl_3_ solvent signals (δ_H_ 7.25 for ^1^H and δ_C_ 77.23 for ^13^C). Positive ion HRESIMS data were obtained with a Micromass Q-ToF equipped with leucine enkaphalin lockspray, using *m*/*z* 556.2771 [M + H]^+^ as a reference mass. For column chromatography, silica gel (Merck, 70–230 mesh ASTM) and Sephadex LH-20 (0.25–0.1 mm, Pharmacia, Piscataway, NJ, USA) were used. Precoated silica gel 60 F-254 plates (Merck, Darmstadt, Germany) were used for TLC.

### 3.2. Biological Materials

The marine ascidian *Didemnum* species was collected in the Mangrove located in Nabq/Sharm El-Sheikh on the Egyptian Red Sea coast at depth (~1 to 2 m) during July 2013. A complete description of ascidian as previously mentioned [13]. The ascidian forms thin (2–3 mm) deep orange colored sheets around the pneumatophores of the mangrove plant *Avicennia marina*. The color of the ascidian fades on preservation in formalin. *Didemnum* species share many characteristics common among colonial tunicate species. Characteristics such as colony shape and color, where the colony grows, zooid structure, and spicule shape have been used previously to separate and identify different *Didemnum* species. *Didemnum* species are characterized by: many small zooids 1–2 mm in length, embedded in a sheet-like, gelatinous matrix called a tunic or test. White, calcareous, stellate spicules are embedded within the tunic’s surface among zooids that contain individual oral siphons while atrial siphons discharge into a common cloacal aperature maintained in deep crevices within the colony [31,32]. Spicules measure 40 μm in diameter on average, but can reach 100 μm. This combination of spicules and zooids give the tunic’s surface an overall appearance described as “small, white dots and pinhole-sized pores”. Descriptions of *Didemnum* sp. colony shape include long, ropey or beard-like colonies; low, undulating mats with short appendages that encrust or drape; sponge-like colonies. However, it should be noted that *Didemnum* sp. colony shape changes with age. Young *Didemnum* sp. colonies usually present as thin mats but as the colony matures, irregular lobes are formed, thereby greatly increasing surface complexity. A voucher specimen, measuring 2.5 × 3.0 cm was deposited in the Red Sea Invertebrates collection of the Department of Pharmacognosy, Faculty of Pharmacy, Suez Canal University under the registrationcode 13DY31.

### 3.3. Extraction and Purifications of Compounds **1** and **2**

The fresh ascidian (180 g wet weight) was extracted with a mixture of CH_2_Cl_2_:MeOH (1:1). The crude extract was defatted with petroleum ether. The defatted extract was partitioned between CH_2_Cl_2_ and H_2_O. The CH_2_Cl_2_ extract (5.8 g) was subjected to VLC using *n*-hexanes:EtOAc and EtOAc:MeOH gradients to afford four fractions: F-1 (190 mg, *n*-hexanes 100%), F-2 (1.35 g, CHCl_3_ 100%), F-3 (1.95 g, EtOAc 100%), and F-4 (1.85 mg, MeOH100%). Fraction F-3 (1.95 g, EtOAc 100%) was chromatographed on a Sephadex LH-20 column (0.25–0.1 mm; 150 g × 50 × 3 cm) using MeOH as an eluent. Fractions of 25 mL were collected and monitored by TLC to obtain five subfractions, F-3A (235 mg), F-3B (170 mg), F-3C (211 mg), F-3D (272 mg), and F-3E (425 mg). Subfractions F-3B (380 mg) and D-3C (265 mg) were subjected separately to silica gel columns (0.04–0.063 mm; 50 g × 50 × 1.5 cm) using CHCl_3_:MeOH gradient elution. The collected fractions were monitored by TLC using anisaldehyde/H_2_SO_4_ as spraying agent to afford impure compounds **1** (Subfraction F-3B) and **2** (Subfraction F-3C), respectively. Purification of the compounds was achieved on Sephadex LH-20 using MeOH as an eluent followed by final purification on a semi-preparative HPLC column (RP_18_, 5 μm, ARII Cosmosil, 250 × 10 mm, Waters). The mobile phase used was 40:60 (v/v) CH_3_CN:H_2_O, detected at 220 nm and at a flow rate of 2.0 mL/min to afford **1** (9 mg, colorless oil) and **2** (7 mg, colorless oil).

### 3.4. Biological Evaluation of the Compounds

#### 3.4.1. Evaluation of Antiproliferaive and Cytotoxic Activities

The effects of the compounds **1** and **2** on HeLa cell proliferation and cytotoxicity were evaluated using the sulforhodamine B (SRB) assay [28,29,30]. HeLa cells were grown in Basal Medium Eagle (BME) containing Earle’s salts, 10% FBS and 50 µg/mL gentamycin sulfate. Cells were plated at a density of 2500 cells per well in a 96-well plate and allowed to adhere and grow for 24 h before compounds were added. The compounds were solubilized in DMSO and added to a final DMSO concentration of 1% in both test wells and vehicle controls. The cells were incubated with compounds or vehicle for an additional 48 h. The IC_50s_, the concentrations required to cause a 50% inhibition of cell proliferation, were calculated from the log dose response curves. The values represent the average of 3–4 independent experiments, each conducted in triplicate ± SEM. Cytotoxicity was determined by a cell density lower than that measured at the time of drug addition (dashed line in Figure 3). Paclitaxel was used as a positive control.

#### 3.4.2. Evaluation of the Antimicrobial Activity

The *in vitro* antimicrobial activity was evaluated using disc diffusion method as previously described [33]. A variety of test microorganisms were used including Gram-positive bacteria (*Staphylococcus aureus* ATCC 25923), Gram-negative bacteria (*Escherichia coli* ATCC 25922) and yeast (*Candida albicans* ATCC 14053). Adjusted inoculums of each microorganism, equivalent to a turbidity of 0.5 McFarland standards, were streaked separately using sterile swabs over the surface of Muller Hinton agar plates. Sterile filter paper discs (6 mm diameter) were impregnated with 20 μL of each compound (1000 μg/mL) and applied to the inoculated plates. The plates were incubated at 37 °C for 24 h. Solvent control discs were used to determine any solvent effect. The activity of each compound was determined by measuring the diameter of the inhibition zone in mm. The experiments were performed in triplicate and the mean diameter of each inhibition zone was recorded.

**Didemnaketal F** (**1**). Colorless oil; [α]_D_ −72.8° (*c* 1.5, CHCl_3_); UV (MeOH) λ_max_ (log ε) 252 (3.93) nm; IR *ν*_max_ (film) 3460, 1735, 1720, 1675, 1650, 1070 cm^−1^; NMR data, see Table 1; HRESIMS *m*/*z* 853.4950 (calcd for C_45_H_73_O_15_ [M + H]^+^, 853.4949).

**Didemnaketal G** (**2**). Colorless oil; [α]_D_ −85.7° (*c* 1.5, CHCl_3_); UV (MeOH) λ_max_ (log ε) 258 (3.86) nm; IR *ν*_max_ (film) 3465, 1731, 1718, 1065 cm^−1^; NMR data, see Table 2; HRESIMS *m*/*z* 857.4899 (calcd for C_44_H_73_O_16_, [M + H]^+^, 857.4898).

## 4. Conclusions

In conclusion, investigation of the CH_2_Cl_2_ fraction of the Red Sea marine ascidian *Didemnum* sp. afforded two new spiroketals, didemnaketals F (**1**) and G (**2**). The structures of the compounds were assigned using spectroscopic studies. Compounds **1** and **2** displayed moderate activity against HeLa cells with IC_50s_ of 49.9 and 14.0 µM, respectively. In addition, didemnaketal F (**1**) showed potent antimicrobial activity against *E. coli* and *C. albicans* with inhibition zones of 20 and 24 mm, while didemnaketal G (**2**) showed moderate activity against *C. albicans* with an inhibition zone of 17 mm at a concentration of 100 µg/disc. These results provide further insight into the chemical diversity and biological activities of this class of compounds.
